# Combining Infrared Vein Visualization and Ultrasound Guidance for Central Line Placement in Difficult Venous Access Patients: A Technical Report

**DOI:** 10.7759/cureus.83264

**Published:** 2025-04-30

**Authors:** Samar Patankar

**Affiliations:** 1 Medicine, St. George's University School of Medicine, St. George's, GRD

**Keywords:** central line placement, difficult vascular access, infrared vein visualization, peripheral venous access, ultrasound-guided central venous catheterization

## Abstract

Central venous catheterization (CVC) is an essential critical care procedure with many indications, such as dialysis, medication administration, and hemodynamic monitoring. However, it can be challenging to perform in specific patient groups, particularly in those with obesity or in oncologic patients. The gold standard technique for CVC is ultrasound guidance (USG), which is a superior method to the traditional landmark-based approach as it reduces the rate of complications such as pneumothorax or arterial puncture. Despite being the gold standard, the ultrasound-guided approach may present technical challenges for deeper vessels in certain patients, such as those with significant obesity due to poor acoustic windows. Infrared vein visualization (IVV) is a known technique commonly used for superficial vein mapping and peripheral venous access. This technical report explores the combined use of IVV and USG for CVC placement. It aims to optimize vein localization and reduce procedural difficulties, particularly in patients with anticipated vascular access challenges due to factors such as obesity or prior unsuccessful catheterizations. The proposed theoretical approach involves pre-procedural vein mapping with IVV, followed by real-time USG for catheter insertion, particularly in patients with obesity. Although IVV alone has limited utility in such patients due to its shallow penetration depth, its use as a complementary tool for pre-procedural mapping followed by ultrasound-guided cannulation may enhance CVC placement in this population by improving site selection and reducing failed attempts. Further clinical studies are required to assess the efficacy and cost-effectiveness of this approach.

## Introduction

The term “central venous catheter” (also known as central venous line or central line) refers to an indwelling device that is inserted into a large central vein such as the internal jugular, subclavian or femoral and progressed until the terminal lumen is within the inferior vena cava, superior vena cava or right atrium [1]. Over the decades, central line placement has become an important procedure in the management of critically ill patients for the administration of medications, hemodynamic monitoring, and total parenteral nutrition. Central venous catheterization (CVC) placement has certain risks, such as pneumothorax, arterial puncture, and catheter-related bloodstream infections (CLABSI). While these complications can occur with any technique, their incidence is notably higher when using the traditional landmark-based approach [2]. Therefore, the introduction of ultrasound guidance (USG) has transformed the care of CVC placement by allowing real-time visualization. The advantages of this method include quicker insertion, a reduction in the number of attempts required, the ability to cannulate difficult venous access (DVA) patients, and even a reduction in CLABSI (which increases as the number of attempts increases) [3]. However, USG may have limitations in patients with morbid obesity, as anatomic landmarks are often obscured in such patients, making the approach more technically challenging [3]. Infrared vein visualization (IVV) technology, such as AccuVein (AccuVein Inc., Medford, NY) and VeinViewer (Christie Medical Holdings, Inc., Lake Mary, FL), has successfully been used in peripheral venous cannulation. These devices utilize near-infrared light that is applied to the skin surface. The light is absorbed in the blood vessels by hemoglobin and reflected in the remaining tissues. The system then processes the returned images and displays them in real-time on the skin surface [4]. While IVV has demonstrated improved first-attempt success rates in peripheral venous access, its application in CVC remains limited, despite the potential for enhanced vessel localization [5]. Given the strengths of both modalities, combining IVV for pre-procedure superficial vein mapping with ultrasound for real-time guidance may optimize central line placement, particularly in patients with risk factors for difficult vascular access, such as obesity. The data for IVV use in CVC placement presented in this report is extrapolated from peripheral vein studies, as its current use in CVC placement is investigational.

## Technical report

Materials

**Figure 1:**
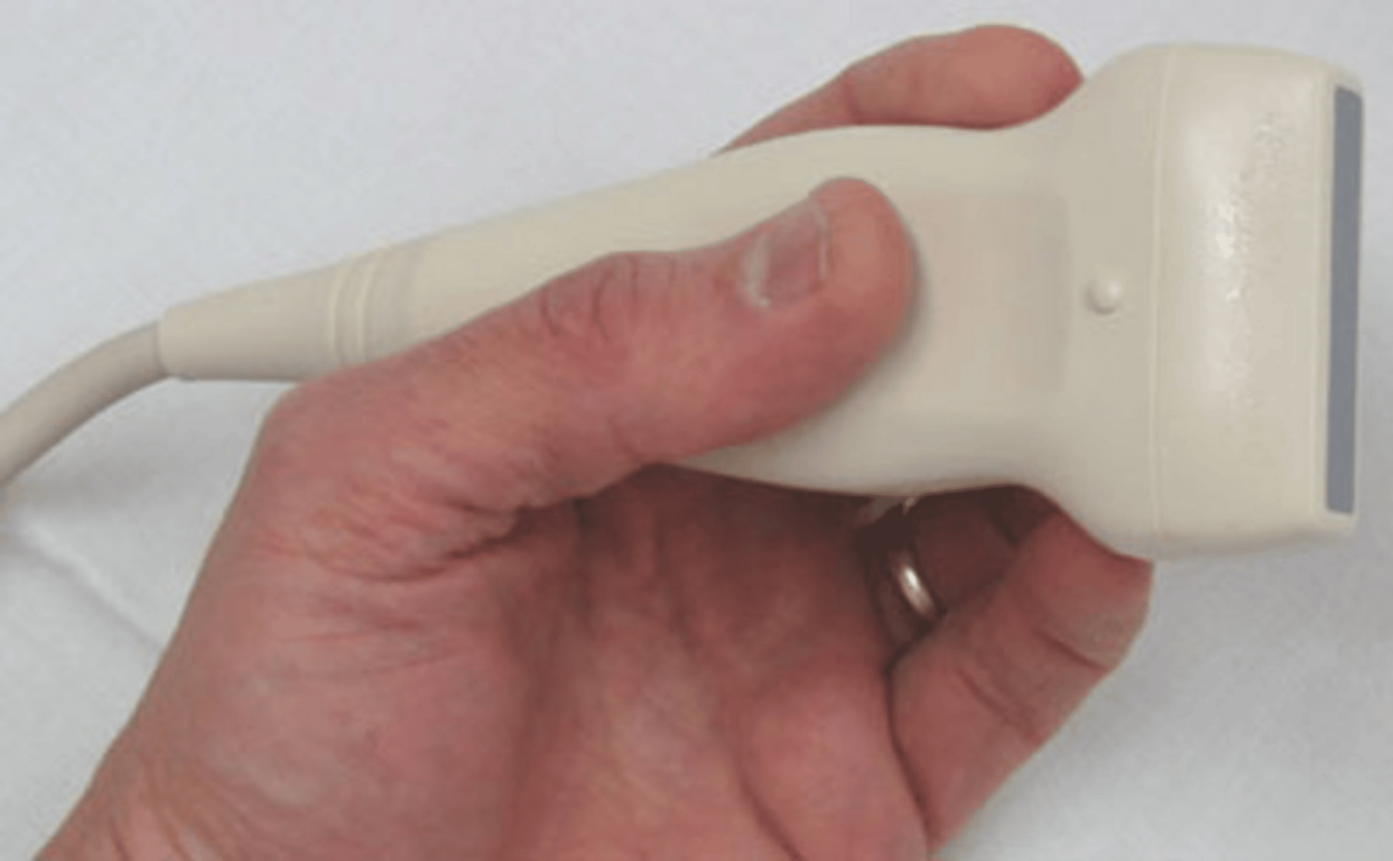
Linear ultrasound probe Image adapted from: Moradmand H. Ultrasound scanner and probe. Available from: https://openverse.org/image/48d9722e-08cf-4492-9875-d7d86e9ba074?q=ultrasound+probe&p=3. Licensed under CC BY-SA 2.0

**Figure 2:**
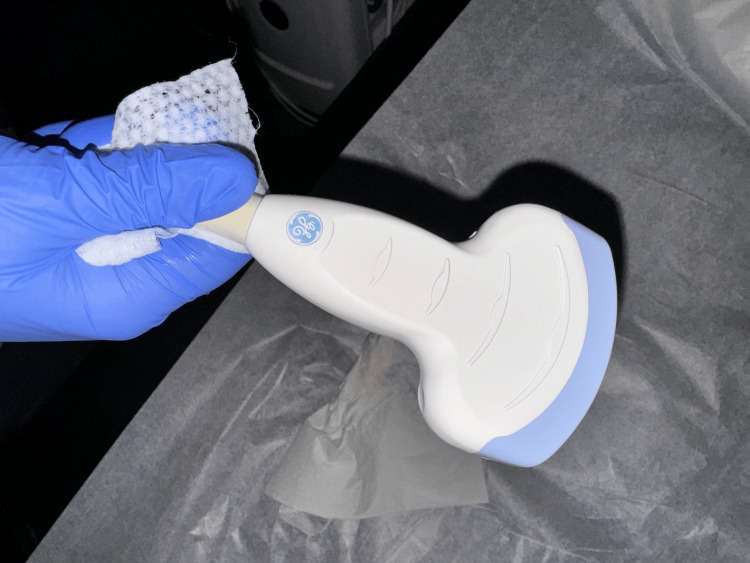
Curvilinear probe Image adapted from: Harrison Keely. Ultrasound probe. Available from https://openverse.org/image/64744439-6b47-4e29-8bdf-f8a3bec414c3?q=ultrasound+probe&p=9. Licensed under CC BY 4.0

**Figure 3:**
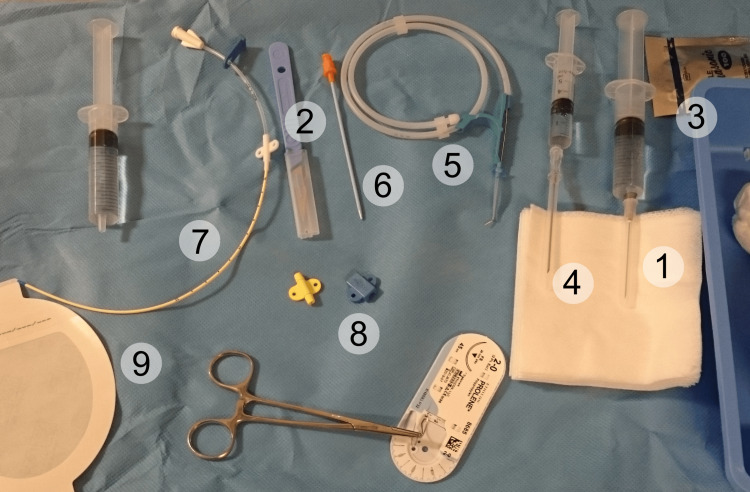
Standard central venous catheterization kit 1. Syringe with local anesthetic. 2. Scalpel. 3. Sterile gel for ultrasound probe. 4. Introducer needle. 5. Guidewire. 6. Tissue dilator. 7. Indwelling catheter. 8. Fasteners and suture. 9. Dressing. Image adapted from: Mikael Häggström. Central Venous Catheter set. Available from: https://openverse.org/image/8422e432-edea-42c3-9c4c-006e51ba32af?q=central+venous&p=11. Licensed under CC0 1.0

**Figure 4:**
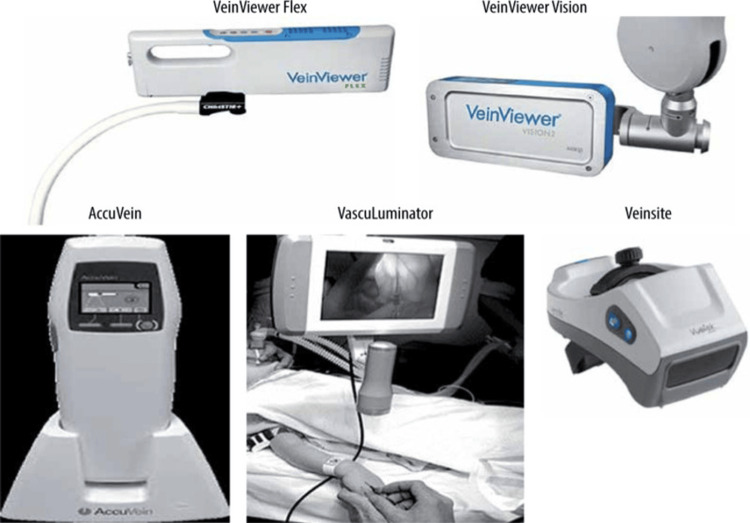
Different types of infrared vein visualization devices Image adapted from Ref. [4]

To implement the combined approach of IVV and ultrasound-guided central line placement, we first ensure that the required equipment is available. An ultrasound machine is required, with the probe choice guided by depth and vein location. The high-frequency linear array probes (10-15 Mhz) are the most commonly used as they have superior resolution and are good for both color and pulse Doppler examination [6]. They are suitable for veins such as the internal jugular. While high-frequency linear probes (Figure 1) remain the preferred choice for real-time needle guidance during central venous cannulation (even in patients with obesity), curvilinear probes (Figure 2) may offer improved depth penetration in certain cases, such as with femoral or subclavian veins. However, their use requires additional operator expertise and may not be routinely used in most clinical settings. We would require a sterile ultrasound probe cover and gel. We also require the standard CVC kit (Figure 3), which must contain sterile gloves, gowns, drapes, antiseptic solution, 1% lidocaine for local anesthesia, introducer needle, guidewire, dilator, an appropriately sized central venous catheter, saline flushes, syringes, sutures, transparent dressing and an IVV device (Figure 4).

Step 1: Patient Selection and Preparation

This combined approach is best suited for patients with risk factors that may complicate vascular access, such as obesity, where the traditional techniques may face challenges. Patients with a history of multiple failed venous access attempts, edema or anasarca, prior thrombosis, or conditions like peripheral vascular disease that cause chronic changes in vasculature are also ideal candidates for this technique. However, certain patients are excluded from this approach. This includes those with extensive venous thrombosis, burns, or skin infections, and those requiring immediate vascular access (such as in life-threatening emergencies where rapid intraosseous or traditional techniques may be faster). Once the patient is identified, consent should be obtained to perform the procedure. It is also necessary to ensure the procedure is performed by a skilled professional with or without a nurse to assist. Once consent is obtained, the room should be cleared of non-essential staff to ensure maximum sterility. A sterile field is necessary to reduce infection risk. The patient should be dressed in a sterile gown, free of any jewelry, and must be connected to a cardiac monitor at the bedside. Patient positioning depends on the target vein. For lines targeting the internal jugular or subclavian, we can place the patient in Trendelenburg, as this reduces the risk of inadvertent air embolism, due to the potential for negative pressure in these veins in the sitting/supine position. For lines targeting femoral veins, the patient should be in a supine position. The patient’s skin tone should be assessed, and the IVV device should be set accordingly. For example, patients with darker skin tones absorb more infrared light; therefore, they would require higher contrast settings. Excess ambient lighting should be dimmed as this can interfere with IVV visualization.

Step 2: Pre-procedure Vein Mapping With IVV

Once positioned, the IVV device is activated, and the wavelength settings are adjusted based on the patient's skin tone. The AccuVein AV500 and VeinViewer Flex are handheld devices and are the most suited for this approach as they allow real-time, dynamic scanning over the skin. We ensure the skin is clean and dry (as moist skin can distort infrared projections) and place the device perpendicular to the skin surface, not in contact with the skin, to avoid pressure artifacts and maintain image clarity. Through this method, we scan the common venous access sites, evaluate vein depth and diameter (which will later be confirmed by ultrasound).

Step 3: Ultrasound-Guided Puncture

**Figure 5:**
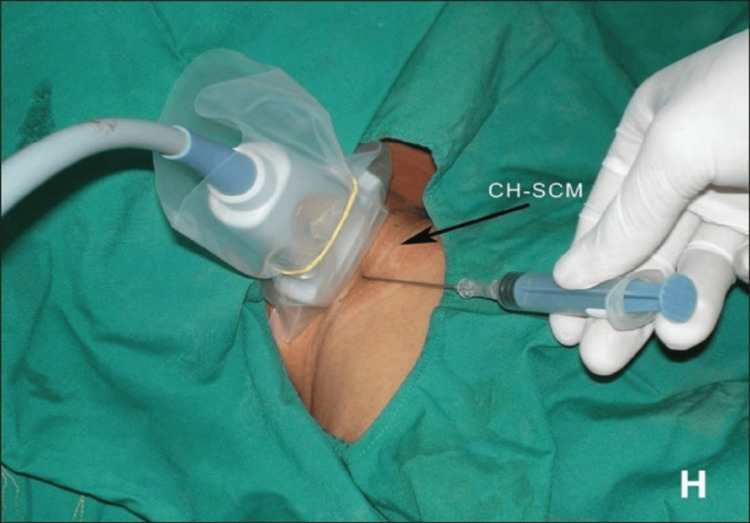
Insertion of central venous catheter in the right internal jugular vein under ultrasound guidance The image shows the position of ultrasound probe and needle with respect to the clavicular head of sternocleidomastoid muscle (CH-SCM). H indicates the head of the patient. Image adapted from Ref. [3].

Once the target vein is identified, the appropriate ultrasound probe is chosen, depending on the clinician's preference. A sterile probe cover is used to maintain the sterile field. The probe must be held over the target site, and cannulation is performed by looking at the ultrasound machine. Compression and Doppler mode are used to confirm venous patency. The probe is positioned in either the short-axis (transverse) or long-axis (longitudinal) view. With the ultrasound beam oriented in a transverse plane perpendicular to the target vessel, the short-axis approach tracks the needle tip as it approaches the target vessel. This approach allows better visualization of adjacent vessels. However, in some cases, it could result in unintended posterior wall puncture of the target vessel. The long-axis approach is performed with the ultrasound beam aligned parallel to the vessel. The advantage of this approach is the ability to visualize the entire needle as it is inserted into the vessel [6]. This approach, however, risks wrong vessel identification and has a higher rate of inadvertent arterial puncture. The needle should be introduced slowly at a 30-45-degree angle under real-time USG (see Figure 5 for positioning).

Step 4: Guidewire and Catheter Placement

**Figure 6:**
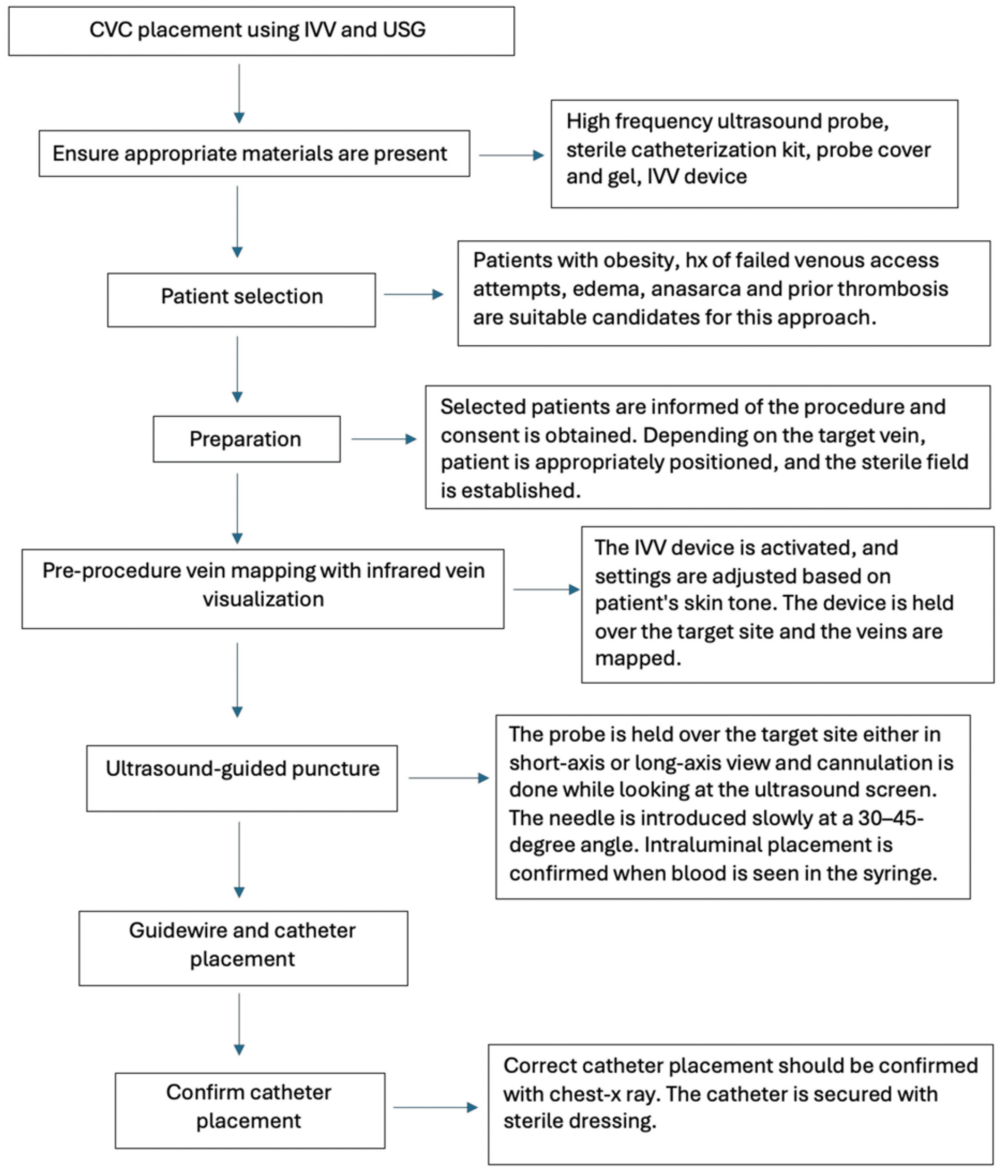
Steps involving the combined approach of infrared vein visualization and ultrasound guidance in central venous catheter placement CVC - central venous catheterization, IVV - infrared vein visualization, USG - ultrasound guidance, hx - history

A guidewire is advanced through the introducer needle under USG. The needle is then removed, leaving the guidewire in place. Ultrasound is used to confirm intraluminal wire placement. Then, the dilator is passed over the guidewire to enlarge the tract, after which the catheter is inserted over the guidewire. At the end, the catheter is secured using a sterile dressing and flushed with normal saline. A chest x-ray should be obtained to rule out pneumothorax and verify catheter tip placement (see Figure 6 for a flowchart on the stepwise approach).

Results

The integration of IVV and USG for central venous catheter placement is expected to enhance procedural success rates and minimize complications, particularly in patients with risk factors for DVA such as obesity, critically ill conditions, or altered vascular anatomy. Studies have shown that real-time USG improves first-attempt success rates for CVC, with reported rates ranging from 71% to 90%, compared to lower success rates and higher complication rates using the traditional landmark technique [6]. Meanwhile, IVV has shown potential in reducing cannulation failure rates in peripheral venous access, with enhanced vein visualization leading to improved vessel selection and reduced procedural time. This theoretical approach of combining IVV for pre-procedural superficial vein mapping with USG for real-time needle insertion may enhance first-attempt success rates (see Table 1 for detailed comparison). While IVV is limited by depth penetration and may not offer substantial benefit in high-risk patients, such as those with morbid obesity, it could assist in identifying superficial collateral veins or anatomical landmarks that may aid access planning. Additionally, the reduction in failed attempts and repeated punctures may lead to a decrease in commonly observed complications such as arterial puncture, hematoma formation, and CLABSI, which are often associated with multiple puncture attempts. Furthermore, this combined technique may also reduce procedure time by streamlining vein localization and minimizing multiple puncture attempts. While peripheral and central venous cannulation are distinct procedures, the demonstrated benefits of IVV in improving peripheral access success rates show potential in supporting central venous access when used adjunctively with ultrasound. It is important to note that the data presented in this report is inferential, since there is no observational data available. The potential benefit of this approach is extrapolated from peripheral venous access studies. Therefore, dedicated studies are needed to validate its utility and effectiveness, specifically in the context of CVC placement. Before standardized guidelines can be considered, preliminary studies are needed to first determine the feasibility of using IVV as an aid in central line placement. If feasibility is established, future prospective studies, such as randomized controlled trials, should focus on validating the clinical effectiveness of the IVV-USG hybrid approach for CVC placement.

**Table 1:** Comparison of the combined approach of infrared vein visualization and ultrasound guidance in central venous line placement with the individual modalities

Aspect	Infrared vein visualization	Ultrasound guidance	Combined approach
Primary care use	Peripheral IV access and vein preservation. Suitable for superficial veins.	Central venous catheter (CVC) placement. Suitable for deeper veins.	Reduces unnecessary CVC placements by improving peripheral access success
Clinical impact	Reduces need for central lines by 25% [6]	Gold standard for CVC with fewer complications	Synergistic effect: 41% reduction in complications vs landmark alone [6]
Procedure time	Faster first attempts (median 14 seconds) [7]	Longer setup but higher accuracy (median 45 seconds) [7]	Infrared accelerates site selection; ultrasound ensures precise cannulation
Success rate	97% improvement in peripheral intravenous access [8]	92%-97% success in CVC placement [8]	Reduces PICC placements by 25-30% when used together [8]
Key advantage	Non-contact vein mapping preserves vasculature	Direct needle visualization prevents complications	Combines speed of infrared with safety of US
Limitation	Limited to superficial structures	Requires trained operators	Requires dual equipment availability and cross-training
Visualization time	Projected vein map on skin surface	Real-time internal anatomy imaging	Infrared for initial assessment + ultrasound for needle tracking

## Discussion

Combining infrared and ultrasound technologies for central venous catheter placement in patients with risk factors for difficult vascular access offers a potential synergistic approach to improve procedural success rates, reduce complications, and enhance patient safety. The traditional landmark-based method of inserting CVC relies heavily on the knowledge of anatomic structures and palpation of arteries next to the veins. Such techniques cannot account for anatomic variations from the normal anatomy, which are described in a relevant proportion of patients for the internal jugular vein, the subclavian and femoral vein [9]. In such cases, ultrasound can easily visualize the anatomic structures and help avoid unintended arterial puncture or unsuccessful cannulation [10]. However, in patients with obesity, image quality and needle-tip visualization may be suboptimal, potentially increasing the difficulty of the procedure. IVV is a non-invasive technique that is used in superficial vein mapping and can serve as a complementary tool. However, because infrared devices are limited to visualizing superficial veins, they are not sufficient as a standalone tool for the deeper vessels that are targeted in CVC. Therefore, the combined approach of using IVV and ultrasound-guidance for CVC placement represents a novel technique that may offer potential benefits. Sekiguchi et al. conducted a crossover simulation study comparing near-infrared visualization with ultrasound in medical students performing peripheral venous access, showing improved vein identification with IVV in novices [7]. While this does not directly translate to CVC, it highlights IVV’s potential to enhance anatomical visualization, which, along with proper supervision, can be used for training purposes for those still learning USG. While infrared mapping is limited by its inability to visualize deep structures such as central veins, it may assist in identifying overlying superficial venous anatomy during pre-procedural planning. However, despite these advantages, specific limitations exist in the combined IVV + USG approach. Firstly, IVV is primarily designed for superficial veins and is not suggested by manufacturers or supported by current evidence for imaging deep central veins, such as the internal jugular, subclavian, and femoral veins. Therefore, it cannot be used as a standalone. Future advancements in infrared imaging technology are needed to expand its role, potentially allowing for deeper vein visualization and broader applications in vascular access. Additionally, operator proficiency is crucial for successfully integrating both techniques, which may necessitate additional training. Also, the aim of this report was to provide theoretical insight rather than presenting new data. Therefore, additional clinical studies and practical assessments are necessary to determine the true impact of this combined approach on patient outcomes, since the report lacks data.

## Conclusions

The integration of IVV with USG may have utility in CVC, particularly in patients with risk factors for difficult vascular access. A comprehensive map of both superficial and deep veins can be obtained by using IVV for pre-procedural vein mapping and ultrasound for real-time guidance, respectively. This may improve procedural efficiency, reduce the number of failed attempts, and minimize complications such as arterial puncture and CLABSI. Given that real-time ultrasound remains the gold standard for CVC placement, further research should focus on quantifying the added benefit of IVV, particularly in terms of patient outcomes, success rates, and complication reduction. This technique could become a valuable addition to existing practices in vascular access, pending validation from randomized controlled studies. 
